# Occurrence, Molecular Serogroups, Antimicrobial Susceptibility and Identification by MALDI-TOF MS of *Listeria monocytogenes* Isolated from RTE Meat Products in Southern Poland

**DOI:** 10.3390/foods13182950

**Published:** 2024-09-18

**Authors:** Renata Pyz-Łukasik, Anna Piróg-Komorowska, Agata Policht

**Affiliations:** 1 Department of Food Hygiene of Animal Origin, Faculty of Veterinary Medicine, University of Life Sciences in Lublin, Akademicka, 12, 20-033 Lublin, Poland; 2 Department of Veterinary Hygiene, Provincial Veterinary Inspectorate in Krakow, Brodowicza, 13b, 30-965 Kraków 69, Poland

**Keywords:** *L. monocytogenes*, RTE meat products, antimicrobial susceptibility, MALDI TOF MS

## Abstract

*L. monocytogenes* is considered one of the most dangerous foodborne pathogens. This study aimed to determine the occurrence of *L. monocytogenes* in RTE meat products from southern Poland, including serogroups and antimicrobial susceptibility, and to assess the usefulness of MALDI-TOF MS as a tool for identifying *L. monocytogenes*. A total of 848 production batches of RTE meat products were analyzed for *L. monocytogenes*. All *L. monocytogenes* isolates were serotyped using the multiplex PCR method, tested for antimicrobial susceptibility using the disk diffusion method and identified using the MALDI-TOF MS method. *L. monocytogenes* was detected in 52/848 batches of RTE meat products (6.13%). The isolates belonged to four serogroups: 17/52 (33%) isolates to IVb; 15/52 (29%) isolates to IIa; 10/52 (19%) isolates to IIc and 10/52 (19%) isolates to IIb. All isolates (52/52) showed susceptibility to the tested antimicrobials. Using MALDI-TOF MS, 10/52 isolates (19.2%) were identified at the level of secure genus identification, probable species identification; 37/52 isolates (71.2%) were identified at the level of probable genus identification; 3/52 isolates (5.8%) were incorrectly identified as *L. innocua*; and 2/52 isolates (3.8%) were not identified. The occurrence of *L. monocytogenes* in RTE meat products was low. Almost half of the analyzed isolates were *L. monocytogenes* of serogroups, which are most often associated with listeriosis in humans in Poland. All isolates showed susceptibility to five commonly used antimicrobials for treating listeriosis. The use of MALDI-TOF MS as a tool for the identification of *L. monocytogenes* indicated its limitations related to the insufficient representation of the pathogen in the reference database.

## 1. Introduction

*Listeria monocytogenes* is a foodborne pathogen responsible for listeriosis in humans. Surveillance of listeriosis in humans focuses on invasive forms of the disease, which take a severe, life-threatening course and most often manifest themselves as sepsis, meningitis or spontaneous abortion [1]. Invasive listeriosis primarily affects elderly people, pregnant women, newborns and individuals with compromised immune systems [1]. However, the infection may also occur in people with no known predisposing factors [2]. According to the European Food Safety Authority, the incidence rate of listeriosis in the population is low (0.62 cases per 100,000) but it is associated with high rates of hospitalization (96%) and mortality (18%), making *L. monocytogenes* one of the most dangerous foodborne pathogens [1]. Data on reported listeriosis cases in 2023 in Poland [https://www.pzh.gov.pl, accessed on 6 September 2024] showed that the incidence rate is comparable to the estimated European level (243 cases; 0.64 per 100,000). The data also indicated an increase in the number of reported cases compared to the previous year (142 cases; 0.38 per 100,000). Furthermore, the analysis of epidemiological data on listeriosis reported in the years 2012–2021 showed that the median incidence was 52.2% higher compared to the previous 5-year period (2007–2011) (0.23 and 0.11 cases per 100,000, respectively) [3]. In the analyzed period, the vast majority of voivodeships in the country reported cases of listeriosis with a hospitalization rate of 97.1% (870/896) and mortality of 8.33–20.83% (in 2020–2021) [3]. In Poland, no listeriosis outbreaks have been reported to date [1,3].

Listeriosis is frequently associated with consuming contaminated ready-to-eat (RTE) food products, with RTE meat products often identified as vehicles of infection [1,4]. There is significant evidence of a high variability in the virulence potential and pathogenicity of *L. monocytogenes* isolates [5,6]. Epidemiological data combined with serotyping results indicate that four molecular serogroups, including IVb (comprising serotypes 4b, 4d and 4e), IIa (comprising serotypes 1/2a and 3a), IIb (comprising serotypes 1/2b, 3b and 7) and IIc (comprising serotypes 1/2c and 3c) were associated with cases of invasive listeriosis in Poland [7]. Of these serogroups, serogroups IVb and IIa were responsible for 90% of the infections, with serogroup IVb responsible for 55.8% of them (*n* = 192/344) [7]. Identifying potential food vehicles of *L. monocytogenes* and the typing of isolates provides information for assessing the risk of exposure to *L. monocytogenes*.

Antimicrobial resistance in pathogenic bacteria is an increasing problem on a global scale [8]. The main strategy for treating infections caused by *L. monocytogenes* is the use of antimicrobials [2]. Resistance in *L. monocytogenes* isolates recovered from various sources, i.e., humans, food and food processing environments, has been reported worldwide [4,9,10,11]. Concerns regarding antimicrobial resistance in *L. monocytogenes* have been increasing for several years and are the subject of research at national and international levels [4,12,13].

MALDI-TOF MS (matrix-assisted laser desorption/ionization time-of-flight mass spectrometry) is standard for identifying pathogenic bacteria within clinical laboratories [14,15]. However, despite its demonstrated efficacy in clinical laboratories, substantial utilization of this method has not been found within food laboratories thus far. MALDI-TOF MS identification is valued for its simplicity, rapidity and cost-effectiveness [14], underscoring its suitability for high-throughput sample applications in routine food control within the food industry.

The current study aimed to determine the occurrence of *L. monocytogenes* in RTE meat products from southern Poland, including serogroups and antimicrobial susceptibility, and to assess the usefulness of MALDI-TOF MS for identifying *L. monocytogenes*.

## 2. Materials and Methods

### 2.1. Food Samples 

Samples were collected from meat processing plants in southern Poland as part of the food safety surveillance program from January 2017 to December 2018. A total of 848 production batches of RTE meat products were analyzed for *L. monocytogenes* according to PN-EN ISO 11290-1:1999/A1:2005 and PN-EN ISO 11290-1: 2017-7 [16,17]. The confirmed *L. monocytogenes* isolates were stored in a brain–heart infusion broth with 15% glycerol at −80 °C until the current analyses. In the present study, these isolates were subject to re-identification according to the procedure described below.

### 2.2. Multiplex PCR

The isolates were cultured on TSYEA medium at 37 ± 1 °C for 18–24 h (Biomaxima, Lublin, Poland). DNA was isolated using the Genomic Mini kit (A & A Biotechnology, Gdańsk, Poland) according to the protocol provided by the manufacturer with the modification of adding 20 µL of lysozyme (10 mg/mL; Merck Sigma-Aldrich, St. Louis, MO, USA) and incubation of the samples for 30 min at 37 ± 1 °C. The identification and classification of *L. monocytogenes* to molecular serogroups was determined with multiplex PCR in accordance with Doumith et al. [18] using primers and the conditions presented in Table 1. The PCR reaction mixture consisted of 5 µL of a nucleotide mixture at a concentration of 2 mM each; 6 µL of a MgCl_2_ solution at a concentration of 25 mM; 5 µL of an enzyme buffer for PCR; 0.5 µL of DNA primers (Genomed, Warszawa, Poland) for the following target genes corresponding to specific serogroups: prs and lmo0737 for IIa, prs and ORF2819 for IIb, prs, lmo1118 and lmo0737 for IIc, and prs, ORF2110 and ORF2819 for IVb (concentration of 10 µM each); 2 µL of thermostable Taq DNA polymerase (concentration of 1 U/µL); and 45.0 µL of DNase- and RNase-free water to obtain the final volume. Moreover, 5 µL of DNA extracted from *L. monocytogenes* was added to the reaction mixture. PCR reagents originated from Thermo Fisher Scientific (Waltham, MA, USA). Gene amplification was carried out in a thermal cycler (Biometra, Göttingen, Germany). *L. monocytogenes* 05CEB424LM, 13CEB102LM (1/2a); 06CEB406LM, 06CEB435LM (1/2b); 06CEB405LM, 13CEB1022LM (1/2c); 06CEB422LM, 16CEL724LM (4b); *L. innocua* ATCC 33090; and *L. ivanovii* ATCC 19119 were used as reference strains (ANSES, Maisons-Alfort, France; Microbiologics, MN, USA).

### 2.3. Determination of Antimicrobial Susceptibility

The antibiotic susceptibility of the isolates was determined by the disk diffusion method. For each isolate, the inoculum with a density of 0.5 McFarland scale was prepared and plated on Mueller–Hinton agar with addition of 5% of defibrinated horse blood and 20 mg/L β-NAD (MH-F) (Biomaxima, Lublin, Poland), and then discs with benzylpenicillin (1 U), ampicillin (2 µg), meropenem (10 µg), erythromycin (15 µg) and trimethoprim–sulfamethoxazole (1.25–23.75 µg) (Biomaxima, Lublin, Poland) were added. Antibiograms were incubated in an atmosphere enriched with 5% CO_2_ at 35 °C for 18 ± 2 h. After the incubation, the growth inhibition zones around the antibiotic discs were measured and analyzed in accordance with EUCAST v. 12.0 [19]. The ATCC 49619 strain of *Streptococcus pneumoniae* was used as a quality control.

### 2.4. MALDI TOF MS Identification

The isolates were cultured on a tryptone soya yeast extract agar medium (TSYEA) (Biomaxima, Lublin, Poland) at 37 ± 1 °C for 24 ± 1 h and processed according to the manufacturer’s instructions using the extraction method. For the analysis, 1 μL of supernatant was spotted on the MSP 96 steel target plate (Bruker, Bremen, Germany), air-dried and overlaid with 1 μL of matrix solution (α-cyano-4-hydroxycinnamic acid) (Bruker Daltonics, Bremen, Germany). The dry plate was then placed in the analysis chamber. Mass spectra were generated and analyzed (*m*/*z* range of 2000–20,000 Da) using an UltrafleXtreme mass spectrometer and MALDI Biotyper (v. 3.1 software package) with a reference database (8468 reference profiles) and the manufacturer’s settings (Bruker Daltonics, Bremen, Germany). For each isolate, 3000 laser shots were collected. Analysis of each sample was performed in triplicate. The Bruker bacterial test standard (Bruker Daltonics, Bremen, Germany) was used for calibration according to the manufacturer’s instructions. Identification results were interpreted according to the manufacturer’s criteria, i.e., 0–1.699—not reliable identification; 1.700–1.999—probable genus identification; 2.000–2.299—secure genus identification, probable species identification; and 2.300–3.000—highly probable species identification. The classification of the analyzed isolates was verified by generating an MSP dendrogram using the MALDI Biotyper (v. 3.1 software package) (Bruker Daltonics, Bremen, Germany).

## 3. Results

### 3.1. Occurrence and Serogroups of L. monocytogenes Isolates

**Figure 1:**
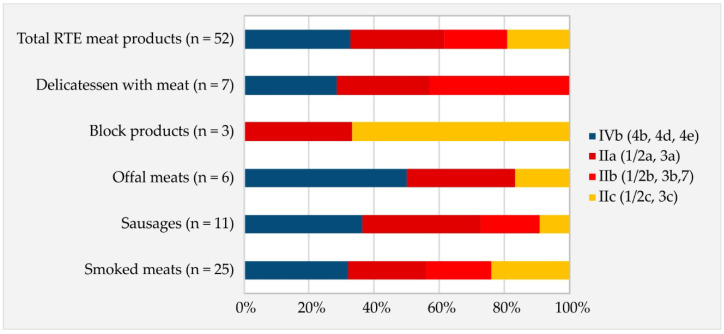
Molecular serogroups of the *L. monocytogenes* isolates in the RTE meat products.

*L. monocytogenes* was detected in 52/848 production batches (6.13%) of RTE meat products (total *n* = 4240 detection units tested, of which positive samples accounted for 2.3%). The isolates were classified into four serogroups: 17/52 (33%) isolates to serogroup IVb (comprising serotypes 4b, 4d and 4e); 15/52 (29%) isolates to serogroup IIa (1/2a and 3a); 10/52 (19%) isolates to serogroup IIc (1/2c and 3c); and 10/52 (19%) isolates to serogroup IIb (1/2b, 3b and 7) (Figure 1). The distribution of individual serogroups in five types of RTE meat products was as follows: serogroups IVb, IIa, IIc and IIb were found in smoked meats and sausages; serogroups IVb, IIa and IIc were found in offal meats; serogroups IIa and IIc were found in block products; and serogroups IVb, IIa and IIb were found in delicatessen with meat (Figure 1).

### 3.2. Antimicrobial Susceptibility of L. monocytogenes Isolates

All *L. monocytogenes* isolates (52/52) showed susceptibility to antimicrobial drugs such as benzylpenicillin (1 U), ampicillin (2 µg), meropenem (10 µg), erythromycin (15 µg) and trimethoprim–sulfamethoxazole (1.25–23.75 µg).

### 3.3. Identification of L. monocytogenes Isolates Using MALDI-TOF MS

**Figure 2:**
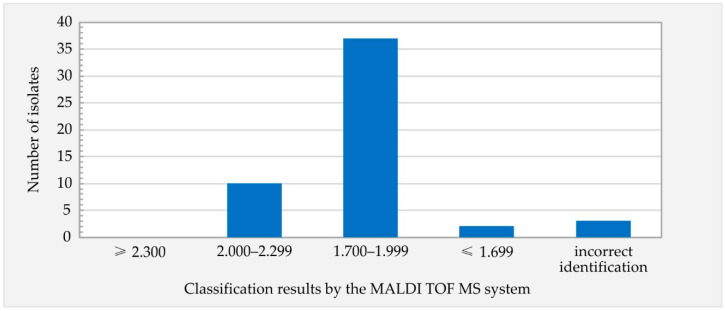
Identification of the *L. monocytogenes* isolates from RTE meat products by MALDI-TOF.

**Figure 3:**
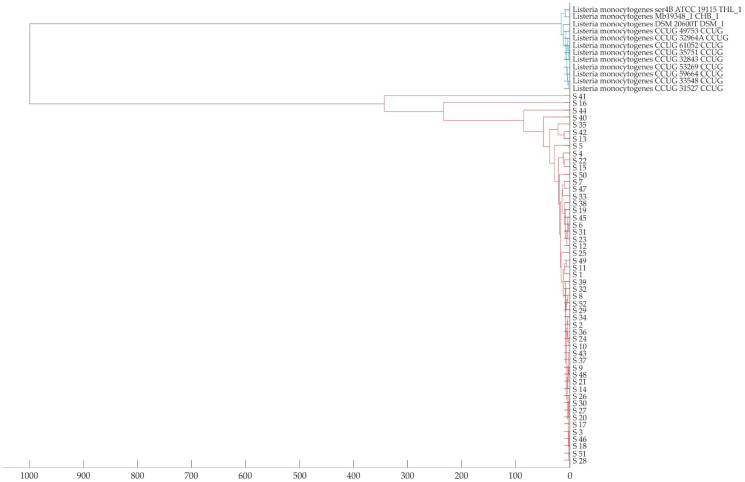
MALDI-TOF MS dendrogram for the 52 analyzed isolates (marked in red) and the reference strains of *L. monocytogenes* (marked in blue). Distance level represents the relative distance used in the clustering analysis.

None of the isolates (0/52) were identified at the level of highly probable species identification (i.e., score values 2.300–3.000); 10/52 isolates (19.2%) were identified at the level of secure genus identification, probable species identification (2.000–2.999); 37/52 isolates (71.2%) were identified at the level of probable genus identification (1.700–1.999); 3/52 isolates (5.8%) were incorrectly identified as *L. innocua*; and 2/52 isolates (3.8%) were not identified (<1.999) (Figure 2). The MSP dendrogram revealed that the mass spectra (MSPs) of the 52 analyzed *L. monocytogenes* isolates formed a distinct branch separate from the MSPs of the reference *L. monocytogenes* strains (Figure 3).

## 4. Discussion

In European Union countries, RTE meat products are subject to official food control for *L. monocytogenes* in accordance with Commission Regulation (EC) No. 2073/2005 [20]. The zero policy for *L. monocytogenes* was adopted with respect to the analyzed RTE meat products [20]. National food control systems are pivotal in ensuring food safety and protecting consumers’ health. In the present study, the official control of 848 production batches of RTE meat products showed that 52 were contaminated with *L. monocytogenes*. The pathogen was found in a wide range of RTE meat products, such as smoked meats, sausages, offal meats, block products and delicatessen with meat (Figure 1). Of the 4240 detection units, 2.3% of the samples were positive for *L. monocytogenes*. This monitoring showed a low occurrence of *L. monocytogenes* in the analyzed RTE meat products. The occurrence of *L. monocytogenes* has been noted in a wide range of RTE meat products worldwide, with high variability in the percentage (%) of positive samples (2.1–64.5%) [1,21,22,23]. The differences in the proportions (%) of positive samples between countries are related to sampling strategy, analytical methods or type of tested food [1,22]. The ubiquitous nature of *L. monocytogenes* presents challenges for controlling and managing this pathogen in food processing plants [21,24]. *Listeria monocytogenes* can enter food processing plants (FPPs) through contaminated raw materials and colonize these environments [21,24]. It is not uncommon to find reports of persistent *L. monocytogenes* isolated over months or years and insufficient disinfection in FPPs [21,24,25]. A large-scale survey concerning *L. monocytogenes* involving twelve European food processing plants producing RTE foods of animal origin, using a harmonized sampling scheme (*n* = 2242), showed that each plant tested positive at least once over the sampling period, with the overall incidence of *L. monocytogenes* reported as being four times higher in meat plants than in dairy plants (*n* = 282; 32% and 8.8%, respectively) [26]. Contamination of the final product with *L. monocytogenes* in FPPs may be related to both contaminated raw material and cross-contamination at the production stage [21,24,25]. The results of this study and literature data clearly show that *L. monocytogenes* remains a constant threat to food safety. Therefore, the potential public health threat posed by *L. monocytogenes* in ready-to-eat food depends on the control and monitoring procedures, which at the production stage should encompass the hazard analysis and critical control points (HACCP), good hygiene practices (GHPs) and sampling procedures to assess adherence to food safety criteria for *L. monocytogenes* [20,27]. Since some listeriosis outbreaks linked to the consumption of ready-to-eat meat products were both associated with social and community events and consumption in households, it is important to raise awareness of the risks associated with the consumption of these types of foods in risk groups [27].

In the present study, four serogroups were identified among the *L. monocytogenes* isolates, i.e., IVb, IIa, IIb and IIc (Figure 1). Isolates belonging to the serogroups most frequently responsible for listeriosis in humans in Poland accounted for 62% of all isolates (IVb 17/52; 32.6% and IIb 15/52; 28.9%), which indicates that RTE meat products from this region of Poland may be a potential source of foodborne listeriosis. The distribution of individual serogroups in the analyzed RTE product category differed from those reported by other authors in the same product category. Maćkiw et al. [13] (*n* = 70) found that the most frequent serogroup was IIa (51%), then serogroups IIc (21%), IIb (14%) and IVb (13%), while Henriques et al. [23] (*n* = 81) showed that the most frequent serogroup was IIb (33%), followed by IVb (27%), IIa (27%), IVa (7%) and IIc (7%). Qualitative and quantitative differences in distribution patterns of the serogroups were also observed between RTE product categories [28]. Serogroup IVb and/or IIa were most frequently reported in RTE meat products, serogroup IIc or IIa in fish products, serogroup IVb or IIa in dairy products and serogroup IIc or IIa in vegetable products [28]. An analysis of the prevalence of individual serogroups linked to producers and ingredients of RTE foods showed that their distribution was related to the prevalence of “intra-plant isolates” of *L. monocytogenes*, which persistently colonized the food processing environments [28].

Prompt implementation of appropriate antimicrobial treatment is crucial to prevent possible complications and long-term sequelae of invasive listeriosis in humans [2]. Penicillin, ampicillin, meropenem, erythromycin and trimethoprim–sulfamethoxazole are used to treat *L. monocytogenes* infections [2]. In the present study, all *L. monocytogenes* isolates were found to be susceptible to these antimicrobials. The susceptibility of *L. monocytogenes* isolates from various sources, including food and food-related sources (100%; *n* = 283) to penicillin, ampicillin and trimethoprim–sulfamethoxazole, and from cases of invasive listeriosis (100%; *n* = 344) to penicillin, ampicillin, meropenem, erythromycin and trimethoprim–sulfamethoxazole, was also reported in Poland [7,12]. However, the resistance of *L. monocytogenes* isolates to the antibiotics analyzed in the current study was also described in other studies from Poland, Italy, Iran and South Africa, with the percentage of isolates resistant to penicillin ranging from 14% to 100% (*n* = 100 and 108, respectively), ampicillin from 44.98% to 83% (*n* = 269 and 70, respectively), meropenem from 10% to 13% (*n* = 108 and 269, respectively), erythromycin from 0.4 to 98.2% (*n* = 283 and 108, respectively), and trimethoprim–sulfamethoxazole from 37.54 to 78.9% (*n* = 269 and 108, respectively) [11,12,13,29,30]. Literature data indicate that the rate of multidrug resistance in *L. monocytogenes* is considered low, although it is characterized by an increasing trend and differences between countries [9,10,31]. Resistance to two or more antibiotics in *L. monocytogenes* isolates (*n* = 68) in Spain significantly increased between 1993 (18.6%) and 2006 (84%), and the average number of antibiotics to which the strains were resistant was lower in 1993 (1.6) than in 2006 (4.2) [9]. In turn, antibiotic resistance, including multidrug resistance among *L. monocytogenes* strains in Germany, was more common than in other European countries and the USA [10]. The emergence of resistance to antimicrobials, including those commonly used to treat listeriosis, highlights the need for continuous surveillance to assess resistance patterns of *L. monocytogenes* over time.

Identification of bacteria by MALDI-TOF MS is based on the comparison of a unique protein profile (spectrum composed of mass-to-charge ratio (*m*/*z*) peaks with varying intensities) specific to a particular bacterial species with a set of mass spectra of the reference database [32]. An alternative method for the identification of *L. monocytogenes* isolates should ensure both the identification of the pathogen at the species level and credible results in the identification process. In the present study, none of the tested isolates achieved a score value ≥ 2300, a threshold indicative of highly probable species identification. Additionally, 10% of the tested isolates were misidentified as *L. innocua* or remained unidentified (Figure 2). These results corroborate prior research, establishing that MALDI-TOF MS, irrespective of the system employed, lacks efficacy in accurately identifying *L. monocytogenes* isolates at the species level [33,34]. This suggests that MALDI-TOF MS possesses inherent limitations, necessitating consideration in its application. MALDI-TOF spectra reflect highly conserved proteins that are minimally affected by environmental conditions [35]. Several studies focused on the identification of microorganisms, including *L. monocytogenes*, using the MALDI-TOF MS method have shown that factors such as the age of the culture or different culture conditions, including the type of medium, do not significantly affect the performance of MALDI-TOF MS when sufficient representation of the tested microorganisms in the database is ensured and pre-analytical and analytical procedures are consistent with those used to create the database [35,36,37,38]. In the present study, adherence to the manufacturer’s sample preparation instructions facilitated the acquisition of high-quality spectra vital for the precise identification of the tested isolates, but these spectra were inadequately represented in the set of reference spectra within the database, as confirmed by the MSP dendrogram (Figure 3). The MSP dendrogram illustrating the phyloproteomic relationship between the tested isolates and the reference strains of *L. monocytogenes* revealed two main separate clades with ingroups. The first main clade included the reference strains of *L. monocytogenes* from the proprietary reference database, while the second main clade included the tested isolates of *L. monocytogenes*. As widely acknowledged, a limited number of mass spectra in the reference database can lead to both subpar species discrimination and misidentification [32], as observed in the current study.

## 5. Conclusions

*L. monocytogenes* is a constant threat to food safety, which highlights the importance of food control and supervision of compliance with cleaning and disinfection procedures in food production environments. RTE meat products may be a potential source of foodborne listeriosis, especially in susceptible populations. Monitoring the antimicrobial susceptibility of *L. monocytogenes* is critical to assessing time-related trends in resistance and ensuring the effective treatment of listeriosis. The future use of MALDI-TOF MS as a tool for identifying *L. monocytogenes* requires expansion of the reference database to ensure the accuracy and reliability of the identification process.

## Figures and Tables

**Table 1:** Primers for molecular serogroup determination using multiplex PCR.

Gene *	Primer Name	Sequence (5′→3′)	Amplicon Size (bp)
*lmo0737*	lmo0737F	AGGGCTTCAAGGACTTACCC	691
	lmo0737R	ACGATTTCTGCTTGCCATTC	
*lmo1118*	lmo1118F	AGGGGTCTTAAATCCTGGAA	906
	lmo1118R	CGGCTTGTTCGGCATACTTA	
*ORF2819*	ORF2819F	AGCAAAATGCCAAAACTCGT	471
	ORF2819R	CATCACTAAAGCCTCCCATTG	
*ORF2110*	ORF2110F	AGTGGACAATTGATTGGTGAA	597
	ORF2110R	CATCCATCCCTTACTTTGGAC	
*prs*	prsR	GCTGAAGAGATTGCGAAAGAAG	307
	prsF	CAAAGAAACCTTGGATTTGCGG	

* Gene amplification was carried out under the following conditions: initial DNA denaturation at 95 °C for 5 min followed by 30 cycles of 94 °C for 1 min, 55 °C for 1 min and 72 °C for 2 min. The final cycle was performed at 55 °C for 2 min and 72 °C for 5 min.

## Data Availability

The original contributions presented in the study are included in the article, further inquiries can be directed to the corresponding author.
